# Positive association between serum calcium-phosphorus ratio and kidney stone prevalence: Analysis of NHANES data (2007–2020)

**DOI:** 10.1097/MD.0000000000048432

**Published:** 2026-04-24

**Authors:** Jian Qin, Xia Liu, Miao Yu

**Affiliations:** a Department of Basic Medicine, Sichuan Vocational College of Health and Rehabilitation, Zigong, Sichuan, China.

**Keywords:** kidney stone prevalence, NHANES, serum calcium-phosphorus ratio

## Abstract

Kidney stones are a common urological disease with a complex pathogenesis influenced by multiple factors. Calcium and phosphorus, as essential minerals in the body, may be closely related to the prevalence of kidney stones. However, studies on the association between the serum calcium-phosphorus ratio and the prevalence of kidney stones remain limited, and systematic analyses are lacking. This study utilized data from the 2007 to 2020 National Health and Nutrition Examination Survey (NHANES), including a total of 34,122 participants. Multivariable logistic regression models were employed, progressively adjusting for demographic characteristics, health status, and dietary factors to assess the association between the serum calcium-phosphorus ratio and kidney stone prevalence. Restricted cubic spline (RCS) analysis was used to explore any potential nonlinear associations between the serum calcium-phosphorus ratio and kidney stone prevalence. Subgroup and interaction analyses were conducted to further investigate the influence of covariates on this association. Among the 34,122 participants, 3207 were diagnosed with kidney stones. The model adjusted for multiple covariates showed a significant positive association between the serum calcium-phosphorus ratio and the prevalence of kidney stones (OR: 1.29, 95% CI: 1.14–1.45, *P* < .001). RCS analysis indicated no significant nonlinear association between the serum calcium-phosphorus ratio and the prevalence of kidney stones (*P* for nonlinearity = 0.558). Subgroup analysis revealed that this positive association remained consistent across multiple subgroups, with no significant interactions observed. Sensitivity analysis further confirmed the robustness of these findings. This study demonstrates a significant positive association between the serum calcium-phosphorus ratio and the prevalence of kidney stones, and this association is linear. Further research is needed to explore the potential role of the serum calcium-phosphorus ratio as a risk marker for kidney stones and to identify possible intervention strategies.

## 1. Introduction

Kidney stones are a common urological disease worldwide, with an increasing incidence trend year by year.^[1,2]^ The causes of kidney stone formation are complex, involving genetic, environmental, dietary, and other factors.^[3,4]^ In recent years, increasing attention has been paid to the relationship between mineral balance in the blood and kidney stone formation, particularly the role of minerals such as calcium and phosphorus.^[5,6]^ However, while the independent effects of calcium and phosphorus have been widely studied, the potential role of the serum calcium-phosphorus ratio as a risk factor remains poorly understood.

Calcium-phosphorus homeostasis is not only a key indicator of bone metabolism but also plays a crucial role in maintaining overall mineral balance, regulating parathyroid function, and supporting cardiovascular health.^[7,8]^ Calcium is a major component of bones and teeth and is involved in vital physiological functions such as nerve conduction, muscle contraction, and blood clotting.^[9,10]^ Phosphorus, on the other hand, is essential for cellular energy metabolism, maintaining cell membrane integrity, and nucleic acid synthesis.^[11,12]^ The balance between calcium and phosphorus is critical for sustaining normal physiological functions. When this balance is disrupted, it can lead to a range of health issues, such as osteoporosis, parathyroid dysfunction, and cardiovascular disease.^[7,8]^ Studies have suggested that disturbances in calcium-phosphorus metabolism may be associated with various metabolic diseases, particularly in the formation of kidney stones, where calcium-phosphorus metabolic imbalances may play a key role.^[13,14]^ However, despite numerous studies on the independent effects of calcium and phosphorus, systematic research on the direct association between the calcium-phosphorus ratio and kidney stone prevalence is still lacking, especially in studies based on large-scale population samples.

Rather than isolated alterations in serum calcium or phosphorus, their relative balance may better reflect systemic mineral homeostasis. The calcium-phosphorus ratio has been proposed as a simple and inexpensive composite marker for identifying disorders of calcium-phosphorus metabolism, particularly in the screening of primary hyperparathyroidism.^[15,16]^ Recent population-based research has further applied the calcium-phosphorus ratio in epidemiological analyses of metabolic diseases, suggesting that it may capture subtle mineral imbalance not fully reflected by either mineral alone.^[17]^ However, its potential relevance to nephrolithiasis has not been systematically investigated in large-scale population studies.

This study, based on data from the National Health and Nutrition Examination Survey (NHANES) from 2007 to 2020, aims to systematically evaluate the association between the serum calcium-phosphorus ratio and the prevalence of kidney stones. By utilizing this large-scale, nationally representative population data, we explore whether the serum calcium-phosphorus ratio serves as an independent risk factor for kidney stone formation, while controlling for other potential confounding factors. This study not only fills gaps in the existing literature but also provides new insights into the prevention and management of kidney stones. The central objective of this study was to determine whether a significant positive association exists between the serum calcium-phosphorus ratio and the prevalence of kidney stones. If confirmed, this finding may identify a new risk marker and offer a scientific basis for kidney stone risk assessment and intervention strategies, as well as provide new perspectives on its pathogenesis and preventive measures.

## 2. Materials and methods

### 2.1. Data source

The data used in this study were sourced from NHANES. NHANES is an ongoing, nationally representative population survey led by the Centers for Disease Control and Prevention (CDC), aimed at assessing the health and nutritional status of the adult and child in the United States. The survey employs a stratified, multistage probability sampling design to ensure that the findings are representative. For this study, data from NHANES between 2007 and 2020 were selected. NHANES data collection includes 3 major components: comprehensive questionnaires, clinical examinations, and laboratory tests. To ensure the representativeness and reliability of the data, we used the sampling weights provided by NHANES to adjust for differences across survey years and population groups. The data analysis utilized merged multi-cycle weights to ensure that the results reflect the overall adult population of the United States. All data were obtained from the publicly available NHANES database, and no personally identifiable information was involved, thus no additional ethical approval was required. To ensure the accuracy and broad applicability of the data, the NHANES data collection and survey design followed strict standardization protocols, and detailed descriptions of all variable definitions and methodologies are available on the NHANES website.^[18,19]^

### 2.2. Study population

**Figure 1.**
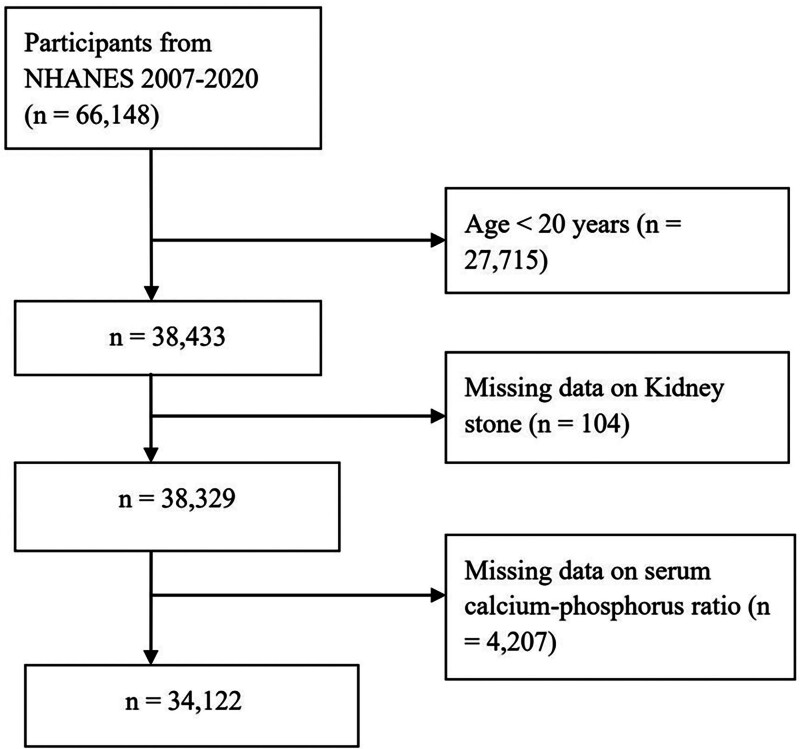
Flowchart of the participant selection process. NHANES = National Health and Nutrition Examination Survey.

The data for this study were obtained from participants in NHANES from 2007 to 2020, with an initial sample of 66,148 individuals. During the screening process, individuals younger than 20 years of age (n = 27,715), those with missing data on kidney stones (n = 104), and those missing data on the serum calcium-phosphorus ratio (n = 4207) were excluded. Ultimately, 34,122 participants were included in the final analysis. Figure 1 presents the participant selection flowchart.

### 2.3. Measurement of serum calcium, serum phosphorus, and calculation of the ratio

This study utilized laboratory data provided by NHANES, with serum calcium and serum phosphorus measured using the automated analysis systems LX20 and DxC800, respectively. Serum calcium measurement: Serum calcium was measured using the ion-selective electrode (ISE) method. The calcium ion concentration was calculated using the Nernst equation, based on the potential change generated by the binding of calcium ions to the ionophore on the electrode surface. Serum phosphorus measurement: serum phosphorus was measured using a timed-rate method. In this process, phosphorus reacts with ammonium molybdate in an acidic solution to form a colored phosphomolybdate complex. The change in absorbance, measured at a wavelength of 365 nm, is directly proportional to the phosphorus concentration. Calculation of the calcium-phosphorus ratio: the calcium-phosphorus ratio was calculated by dividing the serum calcium concentration (mg/dL) by the serum phosphorus concentration (mg/dL). This ratio was used to evaluate the association with kidney stone prevalence.

### 2.4. Definition of kidney stones

In this study, the definition of kidney stones was based on participants’ self-reports from the NHANES health questionnaire. Participants were asked whether they had ever been diagnosed with kidney stones by a healthcare professional. The specific question was: “Has a doctor or other health professional ever told you that you had kidney stones?” Individuals who answered “Yes” were identified as having a history of kidney stones and were classified into the kidney stone group.

### 2.5. Covariates

In the analysis of the association between the serum calcium-phosphorus ratio and the prevalence of kidney stones, several covariates that could potentially influence the results were adjusted for. These covariates covered demographic characteristics, health status, dietary factors, and lifestyle factors, including: age, gender, race/ethnicity (Mexican American, Other Hispanic, Non-Hispanic White, Non-Hispanic Black, and Other Race), education level (less than high school, high school or equivalent, college or above), marital status (married, widowed, divorced, separated, never married, living with a partner), poverty-to-income ratio (PIR), body mass index (BMI), serum creatinine levels, uric acid levels, serum vitamin D levels, daily calcium intake, daily phosphorus intake, daily vitamin D intake, daily dietary fiber intake, daily moisture intake, daily alcohol intake, physical activity (recommended activity and insufficient activity),^[20]^ hypertension (yes/no), diabetes (yes/no), smoking status (never smoked, former smoker, current smoker),^[21]^ and depression status (yes/no). All these covariates were included in the multivariable analysis models to control for their potential influence on the association between the serum calcium-phosphorus ratio and the prevalence of kidney stones.

### 2.6. Statistical analyses

Calcium-phosphorus ratio levels were divided into 4 quartiles: Q1, Q2, Q3, and Q4. Missing values were imputed as follows: mode imputation for categorical variables, mean imputation for normally distributed continuous variables, and median imputation for skewed continuous variables. Categorical variables were presented as percentages and compared using a weighted chi-square test. For normally distributed continuous variables, results were expressed as mean ± standard deviation, and group comparisons were made using a weighted *t*-test. For non-normally distributed continuous variables, median and interquartile range (IQR) were used for description, and comparisons were conducted using a weighted Kruskal–Wallis test. To explore the association between calcium-phosphorus ratio levels and the prevalence of kidney stones, 3 multivariable logistic regression models were constructed. Model 1 was unadjusted for any covariates. Model 2 was based on model 1, with adjustments for age, gender, race, education level, marital status, PIR, BMI, serum creatinine, uric acid, and serum vitamin D levels. Model 3 was adjusted for all covariates. Restricted cubic spline (RCS) analysis was used to explore the nonlinear association between the calcium-phosphorus ratio and the prevalence of kidney stones. Subgroup analyses were conducted to further investigate the association between calcium-phosphorus ratio and kidney stone prevalence in different populations. To examine the potential influence of different covariates on the association between calcium-phosphorus ratio and the prevalence of kidney stones, interaction analyses were performed to test the interactions between calcium-phosphorus ratio and key covariates. All statistical analyses were performed using R software (version 4.2.2; R Foundation for Statistical Computing, Vienna, Austria) and Free Statistics software (version 1.9). A 2-sided *P*-value of <.05 was considered statistically significant.

## 3. Results

### 3.1. Participants and demographic characteristics

This study analyzed NHANES data from 2007 to 2020, including 34,122 eligible participants. Of these, 3207 participants were diagnosed with kidney stones. Serum calcium-phosphorus ratio levels were categorized into 4 groups: Q1 (0.74–2.31) with 8423 participants, Q2 (2.32–2.54) with 8469 participants, Q3 (2.55–2.81) with 8622 participants, and Q4 (2.82–10.10) with 8608 participants. Participants in the Q4 group were more likely to have kidney stones, be older, male, Non-Hispanic Black, have less than a high school education, be widowed, have a lower PIR, a higher BMI, higher creatinine levels, higher uric acid levels, lower serum vitamin D levels, lower daily calcium intake, lower daily vitamin D intake, lower daily dietary fiber intake, lower daily moisture intake, and lower daily alcohol intake. Additionally, they were more likely to have hypertension, diabetes, be former smokers, and not have depression (Table 1).

**Table 1:** Baseline characteristics of the study participants.

		Serum calcium-phosphorus ratio				
		Q1 (0.74–2.31)	Q2 (2.32–2.54)	Q3 (2.55–2.81)	Q4(2.82–10.10)	
Variables	Total (n = 34,122)	(n = 8423)	(n = 8469)	(n = 8622)	(n = 8608)	*P*-value
Kidney stone, n (%)						<.001
No	30,915 (90.6)	7718 (91.6)	7736 (91.3)	7824 (90.7)	7637 (88.7)	
Yes	3207 (9.4)	705 (8.4)	733 (8.7)	798 (9.3)	971 (11.3)	
Age (yr), Median (IQR)	50.0 (35.0, 64.0)	48.0 (32.0, 62.0)	49.0 (34.0, 63.0)	50.0 (35.0, 64.0)	51.0 (37.0, 65.0)	<.001
Gender, n (%)						<.001
Male	16,551 (48.5)	3280 (38.9)	3673 (43.4)	4353 (50.5)	5245 (60.9)	
Female	17,571 (51.5)	5143 (61.1)	4796 (56.6)	4269 (49.5)	3363 (39.1)	
Race, n (%)						<.001
Mexican American	5040 (14.8)	1239 (14.7)	1272 (15)	1276 (14.8)	1253 (14.6)	
Other Hispanic	3648 (10.7)	896 (10.6)	961 (11.3)	902 (10.5)	889 (10.3)	
Non-Hispanic White	13,913 (40.8)	3618 (43)	3429 (40.5)	3465 (40.2)	3401 (39.5)	
Non-Hispanic Black	7301 (21.4)	1584 (18.8)	1714 (20.2)	1902 (22.1)	2101 (24.4)	
Other Race	4220 (12.4)	1086 (12.9)	1093 (12.9)	1077 (12.5)	964 (11.2)	
Education, n (%)						.005
Less than high school	8172 (23.9)	1977 (23.5)	1942 (22.9)	2031 (23.6)	2222 (25.8)	
High school or equivalent	7788 (22.8)	1878 (22.3)	1918 (22.6)	1916 (22.2)	2076 (24.1)	
College or above	18,162 (53.2)	4568 (54.2)	4609 (54.4)	4675 (54.2)	4310 (50.1)	
Marital status, n (%)						.015
Married	18,136 (53.2)	4268 (50.7)	4429 (52.3)	4746 (55)	4693 (54.5)	
Widowed	3871 (11.3)	916 (10.9)	956 (11.3)	972 (11.3)	1027 (11.9)	
Divorced	4362 (12.8)	1039 (12.3)	1121 (13.2)	1072 (12.4)	1130 (13.1)	
Separated	891 (2.6)	247 (2.9)	227 (2.7)	215 (2.5)	202 (2.3)	
Never married	4785 (14.0)	1374 (16.3)	1207 (14.3)	1096 (12.7)	1108 (12.9)	
Living with partner	2077 (6.1)	579 (6.9)	529 (6.2)	521 (6)	448 (5.2)	
PIR, Median (IQR)	2.4 (1.2, 3.8)	2.3 (1.1, 3.7)	2.5 (1.2, 3.9)	2.5 (1.2, 3.9)	2.3 (1.2, 3.7)	.021
BMI (kg/m^2^), Median (IQR)	28.3 (24.5, 32.8)	28.0 (24.0, 32.5)	28.2 (24.3, 32.5)	28.3 (24.5, 32.8)	28.7 (25.1, 33.1)	<.001
Creatinine (mg/dL), Median (IQR)	0.8 (0.7, 1.0)	0.8 (0.7, 1.0)	0.8 (0.7, 1.0)	0.8 (0.7, 1.0)	0.9 (0.7, 1.0)	<.001
Uric acid (mg/dL), Median (IQR)	5.3 (4.4, 6.3)	5.2 (4.3, 6.2)	5.2 (4.3, 6.2)	5.3 (4.4, 6.4)	5.5 (4.6, 6.5)	<.001
Serum vitamin D (nmol/L), Median (IQR)	64.1 (50.5, 72.8)	64.1 (49.3, 75.6)	64.1 (50.3, 73.2)	64.1 (51.3, 72.7)	64.1 (50.8, 69.2)	.027
Daily calcium intake (mg), Median (IQR)	853.0 (586.5, 1100.0)	878.5 (606.5, 1126.2)	859.5 (592.0, 1095.5)	841.2 (583.1, 1095.0)	831.0 (563.9, 1085.8)	.019
Daily phosphorus intake (mg), Median (IQR)	1275.5 (948.5, 1569.0)	1281.0 (958.0, 1581.8)	1276.0 (954.5, 1556.5)	1273.0 (945.0, 1566.4)	1271.5 (937.0, 1570.6)	.596
Daily vitamin D intake (mcg), Median (IQR)	3.7 (1.9, 5.7)	3.9 (2.0, 5.9)	3.8 (1.9, 5.7)	3.6 (1.9, 5.6)	3.5 (1.7, 5.4)	.002
Daily dietary fiber intake (g), Median (IQR)	15.7 (10.6, 20.5)	15.7 (10.8, 20.3)	15.8 (10.7, 20.8)	15.7 (10.8, 20.5)	15.4 (10.3, 20.4)	.693
Daily moisture intake (g), Median (IQR)	2639.9 (1941.6, 3289.8)	2672.4 (1957.0, 3328.9)	2633.9 (1952.9, 3296.7)	2635.7 (1933.3, 3254.4)	2619.6 (1929.6, 3286.4)	.552
Daily alcohol intake (g), Median (IQR)	0.0 (0.0, 8.3)	0.0 (0.0, 8.3)	0.0 (0.0, 8.3)	0.0 (0.0, 8.3)	0.0 (0.0, 8.3)	.075
Physical activity, n (%)						.259
Insufficient activity	2510 (7.4)	580 (6.9)	639 (7.5)	632 (7.3)	659 (7.7)	
Recommended activity	31,612 (92.6)	7843 (93.1)	7830 (92.5)	7990 (92.7)	7949 (92.3)	
Hypertension, n (%)						<.001
No	21,677 (63.5)	5526 (65.6)	5493 (64.9)	5451 (63.2)	5207 (60.5)	
Yes	12,445 (36.5)	2897 (34.4)	2976 (35.1)	3171 (36.8)	3401 (39.5)	
Diabetes, n (%)						.156
No	29,559 (86.6)	7331 (87)	7380 (87.1)	7468 (86.6)	7380 (85.7)	
Yes	4563 (13.4)	1092 (13)	1089 (12.9)	1154 (13.4)	1228 (14.3)	
Smoking, n (%)						<.001
Never	19,145 (56.1)	4805 (57)	4895 (57.8)	4873 (56.5)	4572 (53.1)	
Former	8158 (23.9)	1801 (21.4)	1920 (22.7)	2121 (24.6)	2316 (26.9)	
Now	6819 (20.0)	1817 (21.6)	1654 (19.5)	1628 (18.9)	1720 (20)	
Depression, n (%)						.018
No	31,392 (92.0)	7686 (91.3)	7780 (91.9)	7996 (92.7)	7930 (92.1)	
Yes	2730 (8.0)	737 (8.7)	689 (8.1)	626 (7.3)	678 (7.9)	

BMI = body mass index, g = gram, IQR = interquartile range, PIR = poverty-to-income ratio.

### 3.2. The association between serum calcium-phosphorus ratio levels and the prevalence of kidney stones

**Figure 2.**
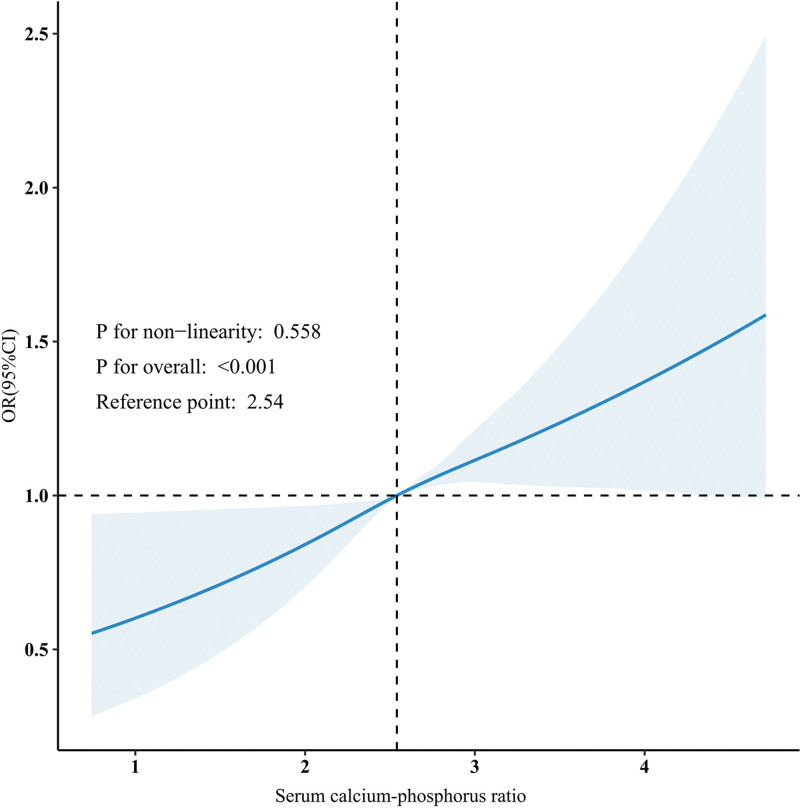
Restricted cubic spline analysis of the association between serum calcium-phosphorus ratio and the prevalence of kidney stones. The reference point (median) is 2.54. CI = confidence interval, OR = odds ratio.

When serum calcium-phosphorus ratio was analyzed as a continuous variable, model 1, which did not adjust for covariates, showed a significant positive association between serum calcium-phosphorus ratio and the prevalence of kidney stones (OR: 1.40, 95% CI: 1.24–1.58; *P* < .001). This association remained significant in both model 2, and model 3 after adjusting for covariates. As serum calcium-phosphorus ratio levels increased, the association became more pronounced, particularly in model 3, where the OR for the fourth quartile was significantly higher than that for the first quartile (OR: 1.32, 95% CI: 1.14–1.53). The trend analysis indicated a significant positive association between serum calcium-phosphorus ratio and the prevalence of kidney stones (*P* for trend < .001; Table 2). After accounting for all potential confounders, the RCS analysis confirmed a significant positive association between serum calcium-phosphorus ratio and the prevalence of kidney stones, with no evidence of nonlinearity (nonlinearity: *P* = .558; Fig. 2).

**Table 2:** The associations between serum calcium-phosphorus ratio levels and the prevalence of kidney stones.

	Model 1		Model 2		Model 3	
	OR (95% CI)	*P*-value	OR (95% CI)	*P*-value	OR (95% CI)	*P*-value
Continuous	1.40 (1.24, 1.58)	<.001	1.31 (1.15, 1.48)	<.001	1.29 (1.14, 1.45)	<.001
Quartile						
Q1	Reference		Reference		Reference	
Q2	1.13 (0.96, 1.33)	.137	1.11 (0.95, 1.31)	.190	1.12 (0.95, 1.31)	.178
Q3	1.16 (1.02, 1.32)	.027	1.11 (0.97, 1.27)	.118	1.11 (0.97, 1.27)	.122
Q4	1.48 (1.28, 1.72)	<.001	1.34 (1.16, 1.56)	<.001	1.32 (1.14, 1.53)	<.001
*P* for trend		<.001		<.001		<.001

Model 1: no covariates adjusted.

Model 2: adjusted for age, gender, race, education, marital status, PIR, BMI, creatinine, uric acid, serum vitamin D.

Model 3: further adjusted for daily calcium intake, daily phosphorus intake, daily vitamin D intake, daily dietary fiber intake, daily moisture intake, daily alcohol intake, physical activity, hypertension, diabetes, smoking, and depression based on model 2.

CI = confidence interval, OR = odds ratio.

### 3.3. Subgroup analyses and interaction effects

**Figure 3.**
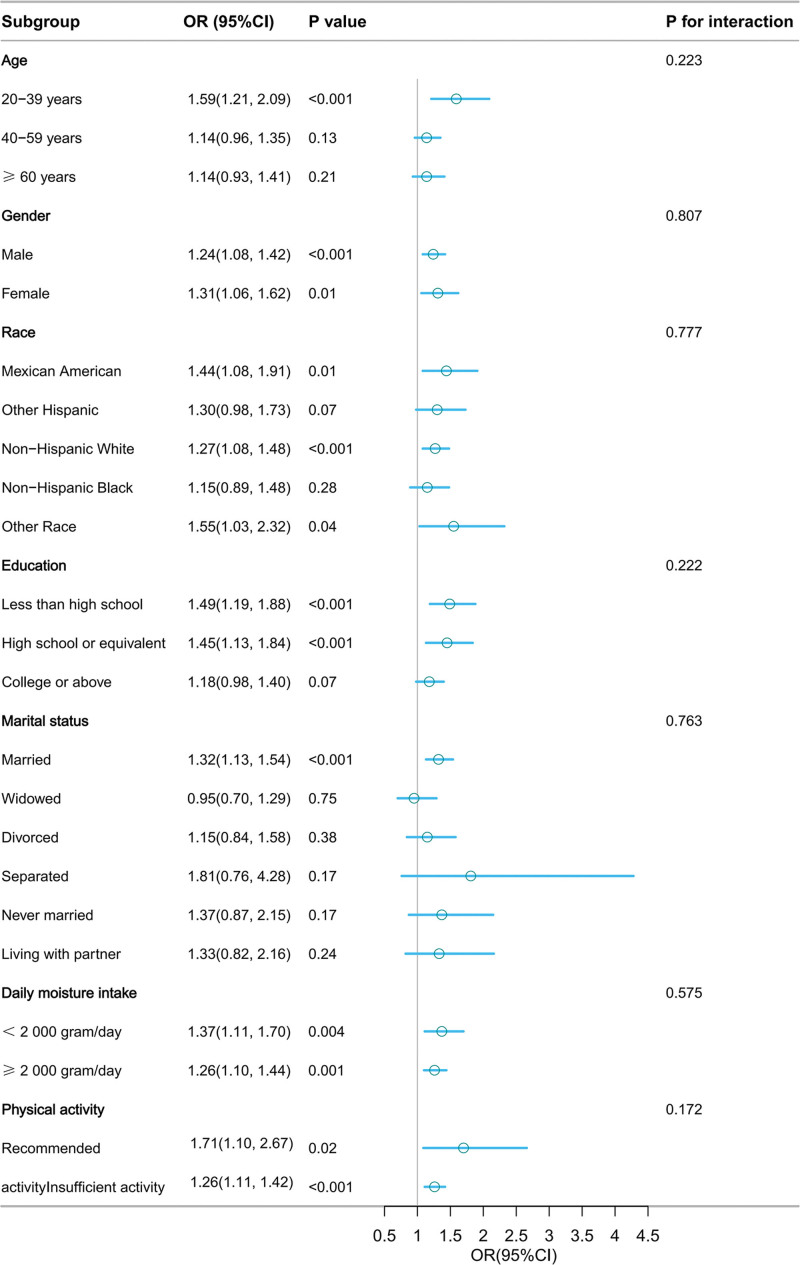
Subgroup analysis and interaction effects for the association between serum calcium-phosphorus ratio and the prevalence of kidney stones. CI = confidence interval, OR = odds ratio.

Subgroup and interaction analyses were conducted based on age, gender, race, education level, marital status, daily moisture intake, and physical activity. The subgroup analysis revealed that the positive association remained consistent across multiple subgroups, with no significant interactions observed (Fig. 3).

### 3.4. Sensitivity analysis results

In the sensitivity analysis, different data processing methods were used to validate the robustness of the results: using the complete dataset (without imputation) and adjusting for multiple covariates, the results showed a significant positive association between the serum calcium-phosphorus ratio and the prevalence of kidney stones (OR: 1.41, 95% CI: 1.16–1.71, *P* < .001); after excluding the top and bottom 2.5% extreme values and adjusting for multiple covariates, the results also showed a significant positive association between the serum calcium-phosphorus ratio and the prevalence of kidney stones (OR: 1.34, 95% CI: 1.16–1.54, *P* < .001; Table 3).

**Table 3:** Sensitivity analysis.

	Model 1		Model 2		Model 3	
Sensitivity analysis methods	OR (95% CI)	*P*-value	OR (95% CI)	*P*-value	OR (95% CI)	*P*-value
The complete dataset, without imputation	1.55 (1.28, 1.87)	<.001	1.41 (1.17, 1.70)	<.001	1.41 (1.16, 1.71)	<.001
After excluding the top and bottom 2.5% extreme values	1.51 (1.30, 1.75)	<.001	1.35 (1.17, 1.56)	<.001	1.34 (1.16, 1.54)	<.001

Model 1: no covariates adjusted.

Model 2: adjusted for age, gender, race, education, marital status, PIR, BMI, creatinine, uric acid, serum vitamin D.

Model 3: further adjusted for daily calcium intake, daily phosphorus intake, daily vitamin D intake, daily dietary fiber intake, daily moisture intake, daily alcohol intake, physical activity, hypertension, diabetes, smoking, and depression based on model 2.

CI = confidence interval, OR = odds ratio.

## 4. Discussion

In this study, we analyzed the association between the serum calcium-phosphorus ratio and the prevalence of kidney stones based on data from NHANES (2007–2020). The results indicated a significant positive association between the serum calcium-phosphorus ratio and the prevalence of kidney stones. Multivariable logistic regression analysis showed that this association remained significant even after adjusting for various potential confounding factors. Specifically, individuals in the highest quartile (Q4) of the serum calcium-phosphorus ratio had a significantly higher prevalence of kidney stones compared to those in the lowest quartile (Q1). RCS analysis further confirmed that this positive association was linear, with no evidence of nonlinearity. The serum calcium-phosphorus ratio and the serum phosphorus-to-calcium ratio are mathematically reciprocal transformations and therefore convey identical statistical information. Accordingly, the choice between them does not affect statistical inference. We selected the serum calcium-phosphorus ratio because it more directly reflects the established role of calcium as the principal component of most kidney stones and enhances clinical interpretability. Existing research suggests that disturbances in calcium-phosphorus metabolism are closely related to the prevalence of kidney stones. Taylor et al’s study included 356 men with newly diagnosed symptomatic kidney stones and 712 healthy men as a control group. Using a prospective nested case-control study design, they analyzed the association between plasma calcium-phosphorus regulatory hormones and the prevalence of kidney stones. The results indicated that calcium-phosphorus metabolic disturbances may be a key factor in the formation of kidney stones.^[13]^ In a study by Zhang et al, 24 patients with calcium stone and 21 non-stone controls were included, with a focus on biomarkers related to calcium-phosphorus metabolism. The study compared the differences in calcium-phosphorus regulatory proteins between the 2 groups and found that the excretion of exosomes carrying calcium-phosphorus regulatory proteins was significantly reduced in the urine of calcium stone patients, further suggesting that abnormalities in calcium-phosphorus metabolism may play an important role in the formation of kidney stones.^[22]^

Beyond the independent effects of calcium and phosphorus, the serum calcium-phosphorus ratio may serve as a more integrated marker of systemic mineral homeostasis. An altered ratio reflects a relative predominance of one mineral over the other and may indicate dysregulation within the tightly regulated calcium–phosphorus–PTH–vitamin D axis.^[23]^ Such systemic imbalance may influence renal mineral handling and alter urinary supersaturation dynamics, thereby modifying crystal formation risk.^[24,25]^ Therefore, the positive association observed between the serum calcium-phosphorus ratio and kidney stone prevalence may reflect broader disturbances in mineral regulation rather than the isolated effect of either calcium or phosphorus alone.

Disturbances in calcium-phosphorus metabolism are closely related to the formation of kidney stones. Calcium and phosphorus are key minerals for maintaining bone health and various physiological functions, and their metabolic imbalance in the kidneys may directly contribute to kidney stone formation. First, an elevated calcium-phosphorus ratio may lead to increased saturation of calcium and phosphorus in the urine, which in turn promotes the formation of calcium oxalate or calcium phosphate crystals. Once these crystals deposit in the renal tubules, they can become the nucleus for kidney stone formation.^[26-28]^ Second, the regulation of calcium-phosphorus metabolism involves several key molecules and hormones, among which fibroblast growth factor 23 (FGF23) plays a central role in regulating serum phosphorus levels. FGF23 inhibits the synthesis of 1,25-dihydroxyvitamin D, reduces the intestinal absorption of phosphorus, and increases renal phosphorus excretion. When serum phosphorus levels rise, FGF23 secretion increases, leading to greater phosphorus excretion and disruption of the calcium-phosphorus balance. This process, accompanied by reduced calcium absorption, may result in increased urinary calcium excretion, thereby promoting the formation of kidney stones.^[13,29-31]^ The calcium-sensing receptor (CaSR) is another important molecule in the regulation of calcium-phosphorus metabolism in the kidneys. When serum calcium levels are elevated, CaSR is activated, reducing calcium reabsorption and increasing urinary calcium excretion. Although this mechanism helps maintain serum calcium levels in the short term, prolonged urinary calcium excretion may promote kidney stone formation.^[32-34]^ Furthermore, disturbances in calcium-phosphorus metabolism may also may influence kidney stone formation through inflammatory and oxidative stress mechanisms. An imbalance in the calcium-phosphorus ratio can trigger local renal inflammatory responses, promoting the release of pro-inflammatory factors such as interleukin-6 (IL-6) and tumor necrosis factor-alpha (TNF-α), which exacerbate kidney damage and promote calcium-phosphorus crystal deposition. At the same time, oxidative stress accelerates renal fibrosis and calcification, increasing the risk of stone formation.^[35-39]^ Thus, an elevated serum calcium-phosphorus ratio influences kidney stone formation through multiple pathways, including increased urinary calcium and phosphorus concentrations, imbalance in calcium-phosphorus regulatory molecules, and the involvement of inflammation and oxidative stress. These mechanisms suggest that maintaining calcium-phosphorus metabolic balance is crucial for kidney health, and that an imbalance in the calcium-phosphorus ratio may be a key driving factor in kidney stone formation.

It is well recognized that urinary calcium and phosphorus excretion are direct determinants of lithogenic risk, as urinary supersaturation of calcium-containing salts is a central step in stone formation.^[40,41]^ Although our study focused on serum measurements rather than urinary indices, systemic disturbances in calcium–phosphorus homeostasis are closely linked to renal mineral handling.^[25]^ Alterations in the serum calcium-phosphorus ratio may therefore reflect upstream regulatory changes that subsequently influence urinary mineral excretion and supersaturation dynamics. However, because urinary biochemical data were not incorporated into the present analysis, future studies integrating both serum and urinary parameters are warranted to further clarify the mechanistic pathway.

Despite this study’s analysis of the association between the serum calcium-phosphorus ratio and kidney stone prevalence using large-scale population data, there are several limitations. First, as a cross-sectional study, it cannot establish a causal relationship, only assessing the association between the calcium-phosphorus ratio and kidney stone prevalence; longitudinal studies are needed in the future to confirm causality. Second, the measurement of the serum calcium-phosphorus ratio was based on a single blood sample, which may not fully reflect long-term calcium-phosphorus metabolism, whereas kidney stone formation is typically a prolonged process. Third, although we adjusted for a variety of covariates in the analysis, there may still be unmeasured confounding factors that could potentially influence the results. In addition, the diagnosis of kidney stones was based on self-reports from participants, which could lead to recall bias or misclassification, thereby affecting the accuracy of the findings. Finally, as the data were sourced from the NHANES database, with the study population primarily consisting of US individuals, the external validity of the results and their applicability to other races and regions require further verification.

## 5. Conclusion

This study, based on large-scale population data analysis, found a significant positive association between the serum calcium-phosphorus ratio and the prevalence of kidney stones, and this association with no evidence of nonlinearity. The balance of calcium-phosphorus metabolism is crucial for the prevention of kidney stone formation, and an elevated serum calcium-phosphorus ratio may be an independent risk factor for kidney stone formation. Future research should further explore the potential role of calcium-phosphorus metabolic interventions and the serum calcium-phosphorus ratio as a risk marker for kidney stones.

## Author contributions

**Conceptualization:** Jian Qin, Xia Liu.

**Data curation:** Jian Qin, Xia Liu, Miao Yu.

**Formal analysis:** Jian Qin, Xia Liu, Miao Yu.

**Methodology:** Xia Liu, Miao Yu.

**Software:** Jian Qin.

**Supervision:** Miao Yu.

**Validation:** Xia Liu.

**Visualization:** Jian Qin.

**Writing – original draft:** Jian Qin, Xia Liu, Miao Yu.

**Writing – review & editing:** Jian Qin, Xia Liu, Miao Yu.
